# Donor-derived acute promyelocytic leukemia presenting as myeloid sarcoma in a transplanted kidney

**DOI:** 10.1038/s41375-020-0903-0

**Published:** 2020-06-10

**Authors:** Risa L. Wong, Megan Ketcham, Trent Irwin, Shreeram Akilesh, Tian Yi Zhang, Jorge D. Reyes, Kerstin Edlefsen, Florencia Jalikis, Pamela S. Becker

**Affiliations:** 1 Division of Hematology, Department of Medicine, University of Washington and Clinical Research Division, Fred Hutchinson Cancer Research Center, Seattle, WA USA; 2 Department of Pathology, University of Washington, Seattle, WA USA; 3 Department of Medicine, Stanford University, Palo Alto, CA USA; 4 Department of Surgery, Division of Transplant Surgery, University of Washington, Seattle, WA USA; 5 Department of Laboratory Medicine, Hematopathology Division, University of Washington, Seattle, WA USA; 6 Department of Pathology, Vanderbilt University Medical Center, Nashville, TN USA

**Keywords:** Acute myeloid leukaemia, Acute myeloid leukaemia

## To the Editor:

Donor-derived malignancies after solid organ transplantation have an estimated incidence of 0.02–0.2%, and donor-derived leukemia is particularly uncommon [1–3]. We present the case of a patient diagnosed with acute promyelocytic leukemia (APL) presenting as myeloid sarcoma in a transplanted kidney allograft. The neoplastic cells were demonstrated to be donor-derived in origin; other organ recipients from the same donor were subsequently tested and had PML-RARα transcripts detected in their peripheral blood.

The patient was a 65-year-old man whose past medical history included nonalcoholic steatohepatitis with cirrhosis, multifocal hepatocellular carcinoma treated twice with radiofrequency ablation and transarterial chemoembolization, severe coronary artery disease with multiple drug-eluting stents complicated by recurrent restenosis, hypertension, type II diabetes mellitus, and obstructive sleep apnea. Three and a half years prior to the current presentation, he underwent liver transplantation with primary non-function of the allograft, requiring a second liver transplant days later. The hospitalization was complicated by shock and repeated ischemic acute kidney injury, resulting in dialysis-dependent end-stage renal disease.

Three years after liver transplantation, the patient received a kidney transplant from a 54-year-old deceased male donor who had died from a cerebrovascular accident. The donor’s initial labs included WBC 11.5 × 10^9^/L, normal hemoglobin, platelet count 27 × 10^9^/L, and INR 1.7. With immediate allograft function, the patient’s creatinine reached a nadir of 1.1 mg/dL. Immunosuppressive agents included basiliximab, tacrolimus, mycophenolate mofetil, and prednisone.

**Fig. 1:**
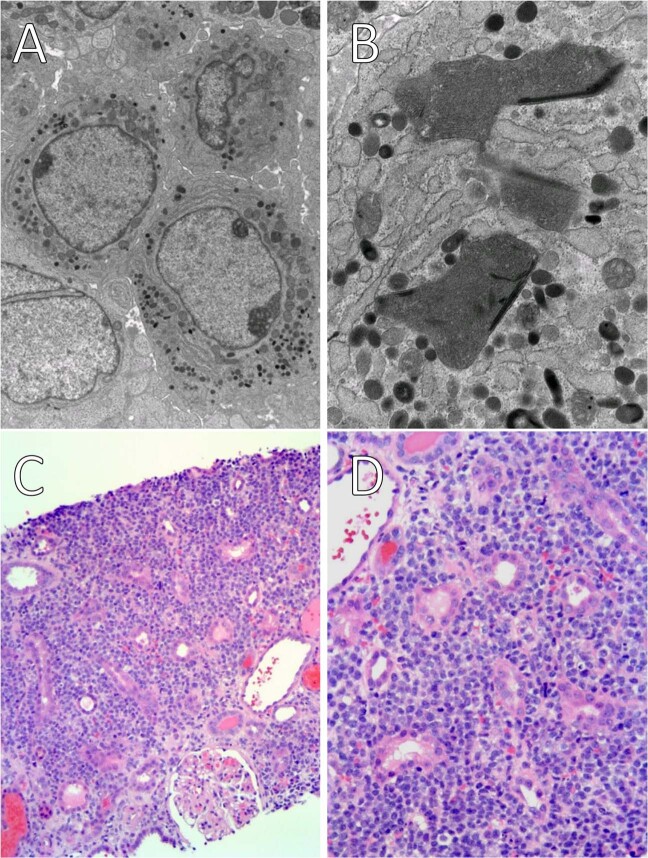
Electron micrographs and light microscopy images of kidney tissue involved by promyelocytes. Ultrastructural examination of the atypical cells (**a**) identified primary and secondary granules, dilated rough endoplasmic reticulum, rare Auer rods (**b**), open nuclear chromatin, and occasionally folded nuclear contours. Light microscopic examination of the second kidney biopsy at low power (**c**) and high power (**d**) demonstrated a diffuse monomorphic infiltrate of mononuclear cells with moderate cytoplasm effacing the kidney parenchyma.

**Fig. 2:**
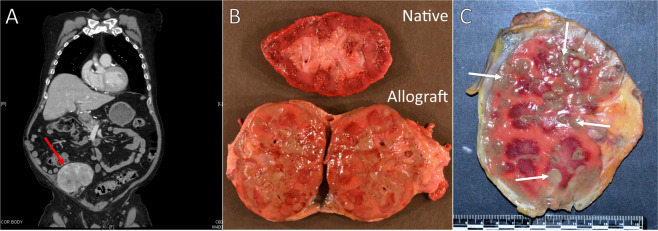
Computed tomography and postmortem examination of abnormal kidney allograft. Computed Tomography (CT) scan of the chest, abdomen, and pelvis (**a**) demonstrated diffuse enlargement and heterogeneous appearance of the allograft kidney (red arrow) with loss of normal architecture differentiating the cortical and medullary parenchyma. Diffuse enlargement and multifocal green-colored masses (white arrows) were present in the allograft kidney (**b**, **c**) on postmortem examination.

Five months after kidney transplantation, the patient experienced intermittent gross hematuria and gradual rise in creatinine to 2.3 mg/dL. Urinalysis showed >50 WBCs/hpf and >20 RBCs/hpf. Biopsy of the allograft kidney initially suggested mild acute cellular rejection based on light microscopy; however, immunofluorescence and electron microscopy showed a monomorphic infiltrate. A myeloid neoplastic process was subsequently favored based on electron micrographs containing cells with an immature myeloid appearance and rare Auer rods (Fig. 1a, b). Immunohistochemistry demonstrated scattered myeloperoxidase positivity, with further characterization hindered by freezing artifact. Therefore, repeat kidney biopsy was performed for full hematopathology evaluation. The abnormal cellular infiltrate was fortunately well-represented in the second kidney biopsy (Fig. 1c, d) with diffuse myeloperoxidase positivity by immunohistochemistry. Flow cytometry revealed an abnormal myeloid blast population comprising 8% of leukocytes and expressing CD117, CD33 (increased), CD34 (variably decreased), CD45 (decreased), and CD13 with no expression of HLA-DR, CD4, CD14, CD15, or CD64. Together, these findings were consistent with myeloid sarcoma. A computed tomography (CT) scan of the chest, abdomen, and pelvis was performed (Fig. 2a) and revealed a diffusely enlarged and heterogeneous allograft kidney with loss of normal architecture; no other significant findings were seen.

Peripheral blood flow cytometry detected a suspicious blast population (0.04% of leukocytes) immunophenotypically consistent with the abnormal myeloid blast population in the kidney. Marrow aspirate flow cytometry identified a similar abnormal myeloid blast population (0.02% of leukocytes). Bone marrow aspirate and biopsy morphology was normocellular with trilineage hematopoiesis. Cytogenetics showed normal male karyotype. We sought additional leukemia cells for molecular and chimerism studies, but further testing of kidney tissue was limited by the sample type, quantity obtained, and prior processing. Given the patient’s pyuria and myeloid sarcoma in the kidney allograft, we hypothesized there could be leukemia cells in his urine. Although not routinely performed, flow cytometry of cells derived from the urine indeed demonstrated the same abnormal myeloid blast population (17% of leukocytes). Chimerism studies on CD117-positive flow-sorted cells from urine resulted as 71% kidney donor and 29% host, consistent with donor-derived origin.

Hours after chimerism results were obtained, the patient developed severe dyspnea in the outpatient setting which progressed to cardiac arrest and pulseless electrical activity. Resuscitation efforts by paramedics were unsuccessful. On postmortem examination, immediate cause of death was attributed to cardiac arrest due to coronary artery disease, with up to 95% stenosis in the left anterior descending coronary artery. The kidney allograft showed multifocal green-colored masses throughout (Fig. 2b, c); histologic sections of these “chloromas” demonstrated kidney parenchyma infiltrated by atypical myeloid proliferation.

A few days later, results of fluorescent in situ hybridization (FISH) testing from the second kidney biopsy returned positive for PML-RARα t(15;17) gene rearrangements consistent with APL, as well as gains of 11q and 21q.

We were concerned about possible transmission to other patients who received transplanted organs from the same deceased donor and reported our case to the United Network for Organ Sharing, discovering that there was a second kidney recipient and a heart recipient from the same donor being followed by other institutions. The second kidney recipient experienced worsening renal function starting 5 months post-transplant; the heart recipient was asymptomatic with no cardiac abnormalities on imaging. We advised that peripheral blood of these patients be sent for RT-PCR testing for PML-RARα transcripts, which returned positive in both patients. The second kidney recipient was initially treated with venetoclax and azacitidine; we advised switching to all-trans retinoic acid (ATRA) and arsenic trioxide (ATO), and the patient was subsequently treated with ATRA alone due to contraindications to ATO. The heart recipient was treated with ATRA and ATO. Both patients are currently in remission with no PML-RARα transcripts detectable by RT-PCR.

In general, reports of donor-derived leukemia after solid organ transplantation typically describe lack of evidence of leukemia in the donor, 2 or more years between transplantation and development of leukemia in the organ recipient, and lack of leukemic disease in other recipients with organs transplanted from the same donor. Thus, it has been hypothesized that nonmalignant hematopoietic stem cells may be carried over with the solid organ, subsequently undergoing malignant transformation in the recipient [4–7]. In contrast, our patient and the other kidney recipient developed renal dysfunction related to myeloid sarcoma within 5 months of transplantation, and the heart recipient tested positive for PML-RARα transcripts within 8 months of transplantation.

One other case of donor-derived leukemia presenting in multiple organ recipients from the same donor has been reported [7]. There is also one other case of donor-derived APL presenting as myeloid sarcoma in a transplanted organ; this presented 6 years after transplantation with no evidence of APL in the other kidney recipient or the liver recipient from the same donor, but they did find postmortem that the deceased donor had evidence of APL [2]. Together, our case and these related cases suggest that the origin of donor-derived leukemia can be hematopoietic cells that have already undergone malignant transformation in the donor, subsequently proliferating in the recipient. In our case, that the neoplastic clone was able to survive and evade clearance in three immunologically distinct host environments, then synchronously expand during the posttransplantation immunosuppression period, is remarkable. Questions remain about how hematopoietic stem cells are transferred from donor to recipient through solid organ transplantation, how donor leukemia cells survive in recipients, and why presentation can occur several months or years after transplantation. A reasonable hypothesis is that pharmacologic immunosuppression may play a role in allowing donor leukemia cells to survive, and that the transplanted organ may have an altered immune microenvironment [2, 8]. These cases have implications on donor selection, and raise the question as to whether rapid sensitive assays for malignancy should be attempted when available.
